# Supplementary material for: Anti-Obesity Efficacy of Pediococcus acidilactici MNL5 in Canorhabditis elegans Gut Model
Source: Int J Mol Sci. 2022 Jan 24;23(3):1276. doi: 10.3390/ijms23031276 (PMC8835910; doi:10.3390/ijms23031276)
Supplement: Supplementary file 1 [file ijms-23-01276-s001.zip › ijms-1557478-supplementary.pdf]

**Isolate Name**	**Protein synthesis inhibiting targeting Antibiotic**				**Cell wall targeting Antibiotics**			**Nucleic Acid targeting Antibiotics**
	Tetracycline (30 µg .L^{-1})	Kanamycin (30 µg .L^{-1})	Clindamycin (30 µg .L^{-1})	Erythromycin (30 µg .L^{-1})	Ampicillin (30 µg .L^{-1})	Vancomycin (30 µg .L^{-1})	Penicillin (30 µg .L^{-1})	Novobiocin (30 µg .L^{-1})
MNL 1	R	R	R	R	R	R	R	R
MNL 2	NR	NR	NR	NR	NR	NR	NR	NR
MNL 3	R	R	R	R	R	R	R	R
MNL 4	NR	R	R	R	NR	R	NR	NR
MNL 5	**R**	**NR**	**R**	**NR**	**R**	**R**	**R**	**R**
MNL 6	R	R	R	R	R	R	R	NR
MNL 8	R	NR	R	NR	R	R	NR	R
MNL 9	R	R	R	R	R	R	R	R
MNL 10	R	NR	NR	R	NR	NR	NR	NR
MNL 11	R	R	R	NR	R	R	R	R
GSC 5	NR	R	NR	R	R	R	R	R
GSC 8	R	NR	R	NR	NR	NR	NR	NR
GSC 10	R	NR	R	NR	R	NR	R	NR
TVL 11	R	R	R	R	NR	R	R	R
TVL 12	R	NR	R	R	R	R	R	R
SKML 3	NR	NR	NR	NR	NR	NR	NR	NR
SKML 4	NR	R	R	R	R	R	R	R
SKML 5	R	R	NR	R	R	R	NR	R
SKML 6	NR	R	R	R	R	R	R	NR
SKML 8	R	NR	R	NR	R	NR	R	R
SKML 10	NR	R	R	R	R	R	R	NR
SKML 11	NR	R	R	R	R	R	R	R
SKML 12	NR	NR	NR	NR	NR	NR	NR	NR
SKML 13	R	R	R	R	NR	R	NR	R
SK SPL 1	R	R	R	R	NR	R	NR	R
SK SPL 6	R	R	NR	NR	R	NR	R	NR
SK SPL7	NR	R	R	NR	R	R	NR	NR
GSC 1	R	R	NR	R	R	R	R	R
GSC 11	R	R	R	NR	R	NR	NR	NR
GSC 12	R	R	R	R	NR	R	R	R
SKML 18	R	R	NR	NR	R	R	R	NR
SKML 20	NR	R	NR	R	NR	R	NR	R

Sup Table S1. Antibiotic susceptibility of probiotic strains. Viability of Lactic Acid Bacteria (LAB) to antibiotics (R: resistant, NR: not resistant).

**Isolate Name**	***Escherichia coli* ATCC 35150**	***Staphylococcus aureus* ATCC 13150**	***Bacillus cereus* ATCC 14579**	***H. pylori* ATCC43504**	***Candida albicans*KCTC 7965**
MNL 1	24	NZ	22	NZ	30
MNL 2	19	10	22	15	20
MNL 3	12	NZ	20	14	21
MNL 4	12	10	10	10	18
**MNL 5**	**28**	**24**	**24**	**18**	**10**
MNL 6	22	20	16	16	28
MNL 8	24	18	14	NZ	13
MNL 9	NZ	18	12	NZ	14
MNL 10	18	NZ	24	12	24
MNL 11	22	21	13	NZ	19
GSC 5	11	NZ	24	NZ	10
GSC 8	16	8	30	12	19
GSC 10	14	12	NZ	12	20
TVL 11	10	18	26	12	22
TVL 12	26	20	NZ	NZ	14
SKML 3	18	21	NZ	NZ	NZ
SKML 4	20	23	9	14	21
SKML 5	NZ	21	8	NZ	NZ
SKML 6	10	18	24	13	24
SKML 8	24	21	16	NZ	14
SKML 10	20	18	NZ	16	21
SKML 11	23	18	NZ	NZ	12
SKML 12	24	NZ	18	NZ	30
SKML 13	22	18	10	10	12
SK SPL 1	22	20	11	NZ	NZ
SK SPL 6	18	18	NZ	NZ	10
SK SPL7	10	10	11	11	21
GSC 1	20	NZ	26	14	20
GSC 11	12	NZ	22	14	21
GSC 12	10	10	14	8	20
SKML 18	NZ	16	18	16	NZ
SKML 20	20	10	24	12	12

Sup Table S2. Antibacterial activity of Lactic Acid Bacteria (LAB) cell free supernatantagainst standard pathogens. (Zone of inhibition measured by millimeter - mm).

Genes	Primers	qPCR condition	Tm (℃)	References
FAT 4	TGGAGGTTTCCTGCTCTCTCA<br>TGGTAAACCATTTGCTGCTGC	95 ℃, 10 min,	80.36	Zhang et al, 2009
FAT 5	CAACTACCATCACACCTTCC<br>CCCGTTCAGTTTCACAGCC	(95 ℃, 15 s, 60 ℃,		Goh, 2017
FAT 7	TTTCCACCACACATTCCCAC<br>TCTTCACTTCCGTGATTGGC	1 min)×40 cycle		Nomura et al, 2010
β-actin	ATCGTCCTCGACTCTGGAGAT<br>TCACGTCCAGCCAAGTCAAG			Nguyen et al, 2007

Sup Table S3. List of primers used for qPCR, genotyping, and sequencing. All sequences are displayed in a 5’ to 3’ orientation.

References

1. Zhang, J., Yang, C., Brey, C., Rodriguez, M., Oksov, Y., Gaugler, R., Dickstein, E., Huang, C.H. and Hashmi, S. (2009). Mutation in *Caenorhabditis elegans* Krüppel-like factor, KLF-3 results in fat accumulation and alters fatty acid composition. *Experimental cell research*, 315(15), pp.2568-2580.
2. Nomura, T., Horikawa, M., Shimamura, S., Hashimoto, T. and Sakamoto, K. (2010). Fat accumulation in *Caenorhabditis elegans* is mediated by SREBP homolog SBP-1. *Genes & nutrition*, 5(1), p.17.
3. Goh, Y.S., 2017. Transcriptional regulators of oxidative stress responses in the nematode *Caenorhabditis elegans* (Doctoral dissertation, University of British Columbia).
4. Nguyen, K., van Die, I., Grundahl, K.M., Kawar, Z.S. and Cummings, R.D., (2007). Molecular cloning and characterization of the *Caenorhabditis elegans* α1, 3-fucosyltransferase family. *Glycobiology*, 17(6), pp.586-599.

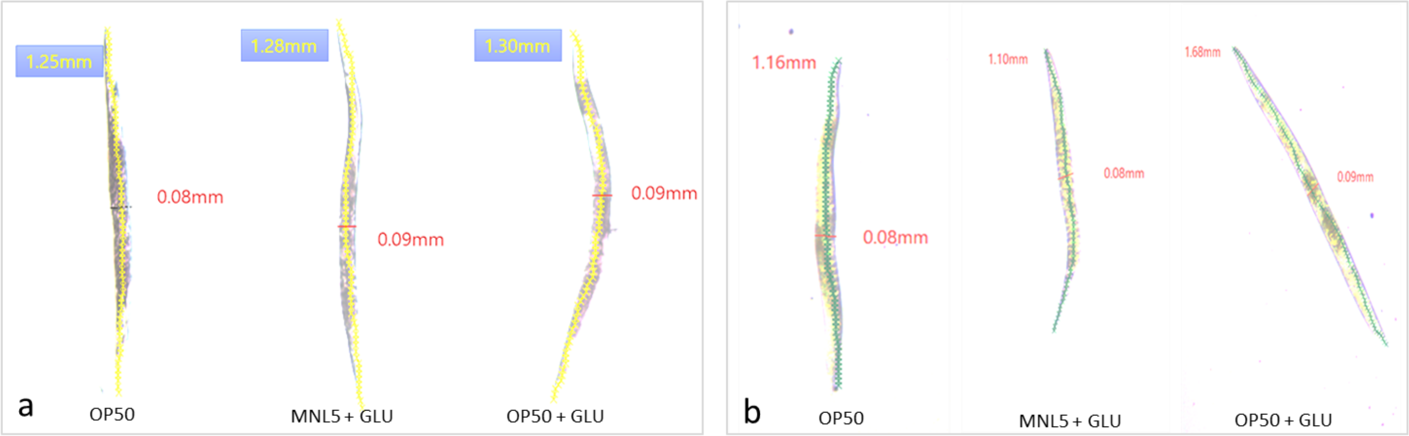

Sup Figure S1. Glucose supplemented diet alteration of *C. elegans* body width and length. a) DAF-2; b) LIU1 model. The treatments such as, OP50; OP50+Glucose; MNL5 + Glucose. Worm size measurements assessed by Olympus SZ 61 zoom stereomicroscope ToupViewTM 3.7 software.

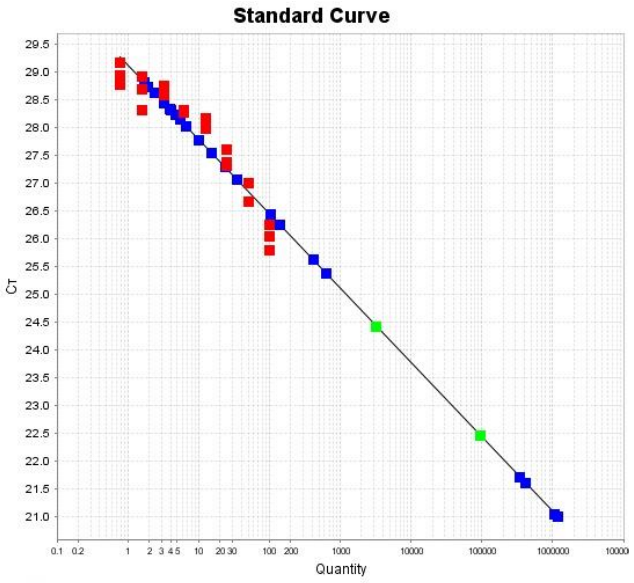

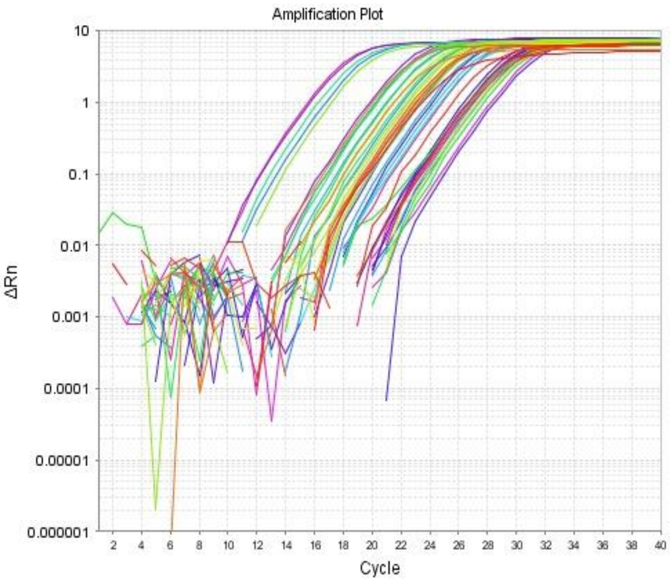

b

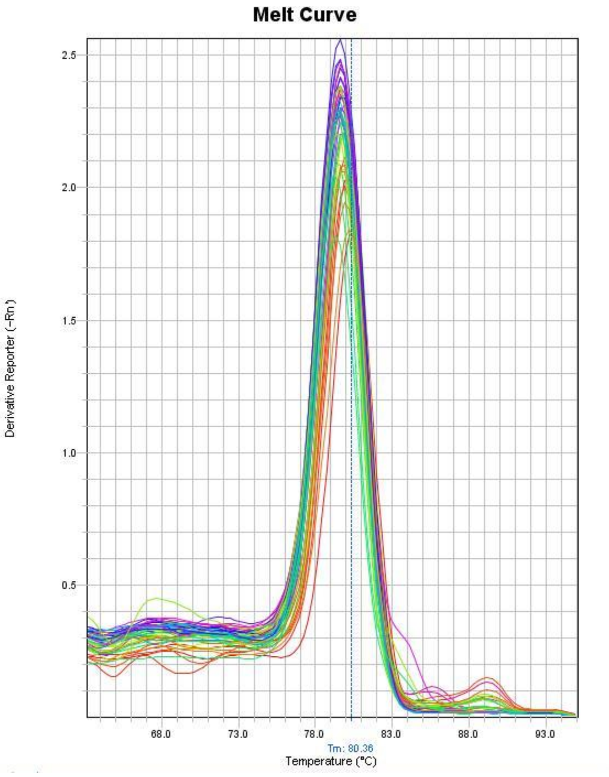

c

Sup Figure S2. a) Standard curve of qPCR using the primer fat genes; b) Amplification plot; c) Melt curve peaks for the detection of fat genes in *C. elegans* models (DAF-2 & LIU1). *C. elegans* concentrations of RNA : 8×10^{1} , 8×10^{1}, 8×10^{0},

8×10^{-1}, 8×10^{-2}, 8×10^{-3}, 8×10^{-4}, 8×10^{-5} ng per reaction. cDNA amounts correspond to $2\times10^{7,}$ 2×10^{6}, 2×10^{5}, 2×10^{4}, 2×10^{3}, 2×10^{2}, 2×10^{1} and 2×10 ° (200 worms), respectively.
